# Acclimation to prolonged hypoxia alters hemoglobin isoform expression and increases hemoglobin oxygen affinity and aerobic performance in a marine fish

**DOI:** 10.1038/s41598-017-07696-6

**Published:** 2017-08-10

**Authors:** Yihang K. Pan, Rasmus Ern, Phillip R. Morrison, Colin J. Brauner, Andrew J. Esbaugh

**Affiliations:** 1 Marine Science Institute, The University of Texas at Austin, Port Aransas, TX USA; 2 Department of Zoology, The University of British Columbia, British Columbia, Canada

## Abstract

Hemoglobin (Hb) multiplicity is common in fish, yet despite its ubiquitous nature, the functional significance is unclear. Here we explore the hypothesis that Hb multiplicity plays a role in hypoxia tolerance using the red drum (*Sciaenops ocellatus*). Red drum is an economically and ecologically important species native to coastal regions and estuaries of the Gulf of Mexico – habitats that routinely experience pronounced hypoxic events. Using a transcriptomic approach, we demonstrate that red drum red blood cells express 7 and 5 Hbα and Hbβ isoforms, respectively. Phylogenetic analysis grouped these isoforms into distinct isoHb clades, and provided evidence of lineage specific expression of particular isoHbs. In normoxia, three isoHbs predominated (Hbα-3.1, -3.2, and Hbβ-3.1). A three-week hypoxia acclimation (48 mmHg) resulted in significant up-regulation of Hbα-2, Hbα-3.2, and Hbβ-3.1, effectively switching the predominantly expressed isoforms. Changes in subunit expression were correlated with a decrease in non-stripped hemolysate P_50_. Similarly, hypoxia acclimation resulted in a 20% reduction in whole animal critical oxygen threshold (P_crit_). Hypoxia acclimation was not associated with changes in gill morphology, hematocrit, or relative ventricular mass. Overall, these data provide support for the hypothesis that Hb isoform switching can provide a physiological benefit to counteract environmental stress in fishes.

## Introduction

Hemoglobin (Hb) multiplicity is a well-documented phenomenon in fish^1–7^. Hb isoforms (isoHbs) in teleosts are classified into three broad categories^8^. Class I Hbs are electrophoretically anodal and exhibit sensitivities to phosphates, temperature, and pH (e.g. *Platessa platessa* and *Platichthys flesus*)^9^. Class II Hbs found in *Oncorhynchus mykiss* ^10^, *Anguilla* sp.^7, 11^, and morays^12, 13^ contain both electrophoretically anodal and cathodal components. The anodal components are similar to Class I Hbs, and are typically characterized by marked Bohr (decreased Hb-O_2_ affinity with a decreased pH) or Root effects (decreased Hb-O_2_ affinity and O_2_ carrying capacity with decreased pH), whereas the cathodal components are relatively insensitive to pH and temperature, and may have intrinsically higher oxygen affinities. Class III Hbs are sensitive to pH but not temperature, and are found in a few active, warm-blooded fish such as tuna^14^. Thus, it has been suggested that fish might be able to respond to environmental challenges such as hypoxia and long-term temperature changes by altering isoHbs^1, 5^.

While Hb multiplicity in fish is well known and wide spread, the physiological implication of multiple isoHbs is unclear. In species such as the striped mullet (*Mugil cephalus*)^6^ and gilt-head bream (*Sparus aurata*)^15^ distinct isoHbs have almost identical functional properties. Conversely, Japanese eel (*Anguilla japonica*) express at least two functionally distinct Hb pools^7^, and acclimation to varying temperatures has also been demonstrated to differentially regulate isoHbs in several species^1, 16, 17^. These findings suggest that Hb multiplicity may confer a physiological advantage under specific environmental conditions, which is supported by similar findings in tetrapod vertebrates^18, 19^.

Of the myriad environmental challenges that fish experience, hypoxia profoundly impacts oxygen uptake. Fish are capable of maintaining oxygen uptake under hypoxic conditions through a variety of mechanisms that include hyperventilation, increased blood perfusion through the gills, and enhanced Hb-O_2_ affinity via modulating the concentration of allosteric modulators in the red blood cell, particularly NTPs. Fish also respond to prolonged hypoxia through gill re-modelling that increases surface area and reduces diffusion distance. This is particularly apparent in species with an interlamellar cell mass, which can be altered to enhance gas transport or reduce ion loss as required. Fewer studies have explored how fish may improve oxygen uptake through the differential regulation of isoHbs. The available evidence in adult fish is mixed^3, 10^; however, more compelling support can be found in early development. Specifically, the Lake Victoria cichlid *Haplochromis* (*Labrochromis*) *ishmaeli* demonstrated differential expression of isoHbs when reared under normoxia and hypoxia^5^. The hypoxia reared fish Hbs also exhibited higher Hb-O_2_ affinities, suggesting that Hb multiplicity may constitute part of a regulatory mechanism that protects oxygen uptake under adverse conditions.

Red drum (*Sciaenops ocellatus*) is an economically important species native to the coastal regions of the Gulf of Mexico (GoM). Like many estuarine species, red drum regularly encounter hypoxia in their natural environment, and this is compounded by the occurrence of wide scale oxygen minimum zones in the northern GoM. Importantly, this species has been shown to exhibit phenotypic plasticity in response to a wide array of environmental perturbations^20–23^. These factors make red drum an ideal species to explore the capacity of a marine fish to acclimate to prolonged hypoxia through phenotypic plasticity, and whether Hb multiplicity may play a role in this plasticity. As such, the first objective was to explore the prevalence of Hb multiplicity in red drum in relation to other teleosts using a combination of transcriptomic and phylogenetic analyses. A second objective sought to explore the effect of prolonged environmental hypoxia on Hb expression, blood oxygen affinity and whole animal critical oxygen thresholds (P_crit_). Additionally, these studies also addressed the potential roles of branchial plasticity in hypoxia acclimation through the analysis of gill surface area and diffusion distance.

## Results

### Phylogenetic analysis

**Figure 1:**
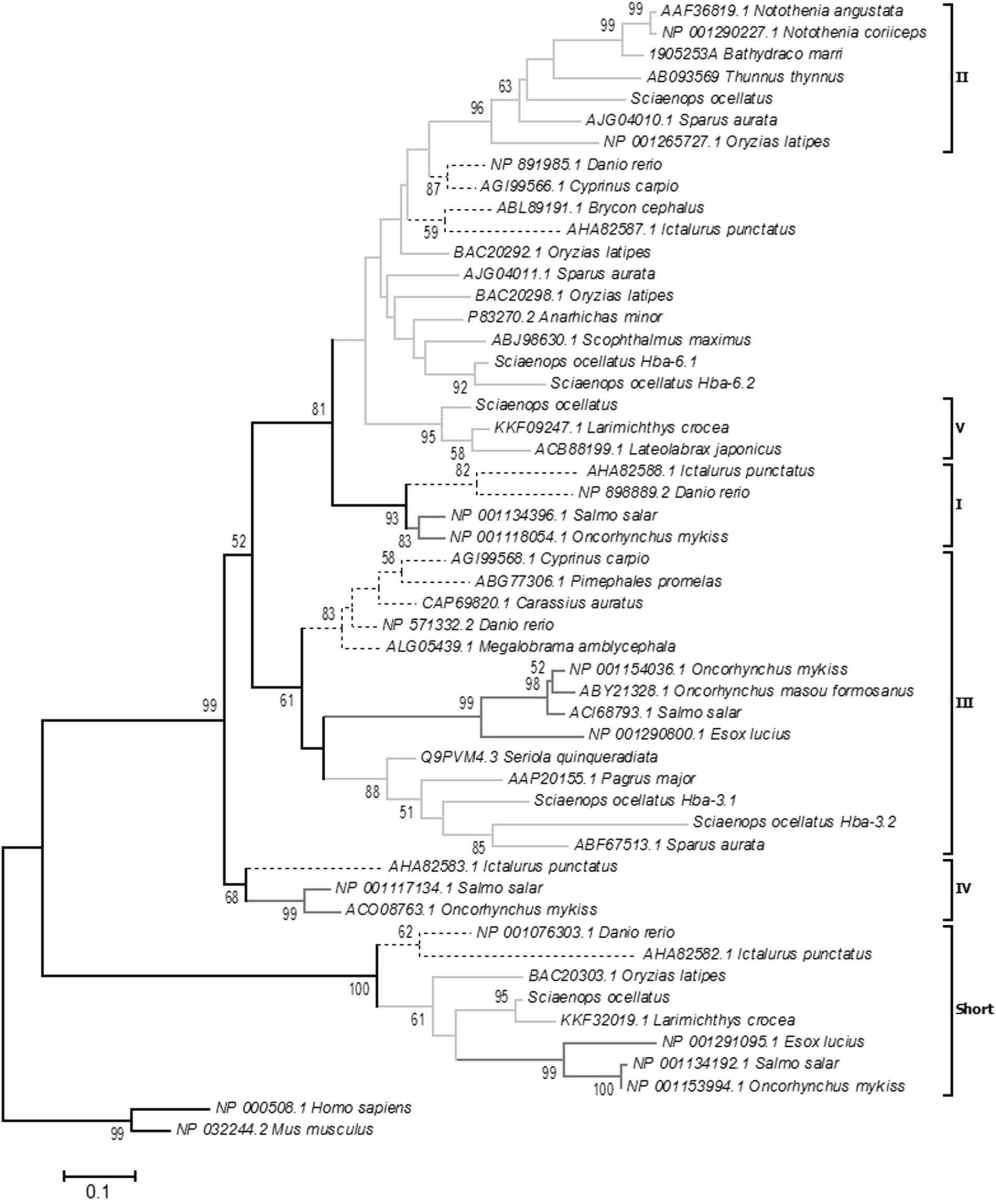
Maximum likelihood analysis showing the phylogenetic relationship for Hbα subunits in teleosts. Different colors represent the species’ respective superorder: light gray = Acanthopterygii; black dotted = Otocephala; dark gray = Protacanthopterygii. GenBank accession numbers are provided within the respective species designations. Numbers represent node bootstrap support. Values below 50% are not shown, which designates a poorly supported node.

A total of 7 Hbα and 5 Hbβ subunits were identified from a red drum red blood cell, gill and intestinal RNA transcriptome. A maximum likelihood phylogeny analysis grouped the Hbα subunits into six well supported clades (Fig. 1). Aside from the previously described 5 groups (I, II, III, IV, short; see discussion), an additional group was identified that did not align with the existing nomenclature and was named V to accommodate the three existing sequences. To provide further evolutionary insight into the multiplicity of Hbα subunits across fish lineages we mapped fish superorder onto the phylogenetic tree. Interestingly, only two Hbα clades (III and Short) contained the derived Acanthopterygii as well as the more basal Otocephala and Protacanthopterygii. Conversely, clades I and IV did not contain Acanthopterygii species, while II and V were exclusive to the Acanthopterygii. The red drum Hbα isoforms fell into clades II, III, V and Short. Also notable was a loosely associated group of isoforms from Otocephala and Acanthopterygii that could not be accurately resolved, yet contained two additional red drum isoforms. These sequences were named Hbα-6.1 and Hbα-6.2.

A similarly well supported phylogeny was obtained for the Hbβ subunits, which identified 5 distinct clades (data not shown). In this case the majority of clades contained all three superorders. The lone exception was clade II, which only contained two sequences and was not expressed by red drum.

### Hemoglobin multiplicity

**Figure 2:**
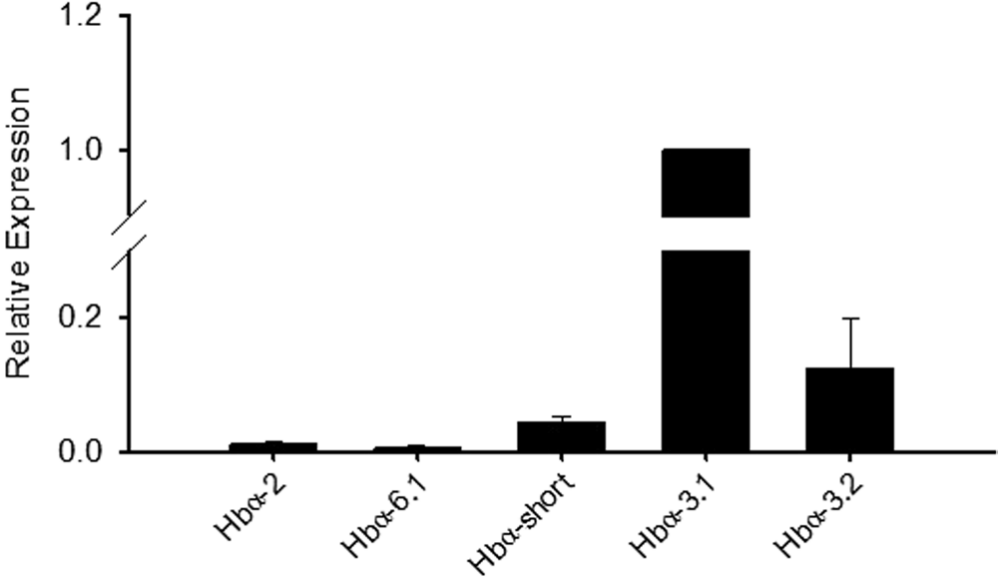
Relative mRNA expression of the five detectable Hbα subunits in red blood cells of *S*. *ocellatus*, as determined by real-time RT-PCR. Ef1α served as an internal control, and all expression value were calculated relative to the Hbα-3.1 subunit. All values are mean ± S.E.M; *N* = 7.

Of the 12 Hb subunit isoforms identified in the red drum transcriptome, 10 exhibited detectable expression via real-time PCR. The relative expression values of the detectable Hbα subunit isoforms revealed that Hbα-3.1 was the predominantly expressed isoform, with the Hbα-3.2 isoform the second most expressed at 12 ± 7% relative abundance (Fig. 2). While all 5 Hbβ subunit isoforms exhibited detectable mRNA expression, the Hbβ-3.1 isoform exceeded all other isoforms by over 6 cycle thresholds (*i*.*e*. 64-times greater expression; data not shown).

**Figure 3:**
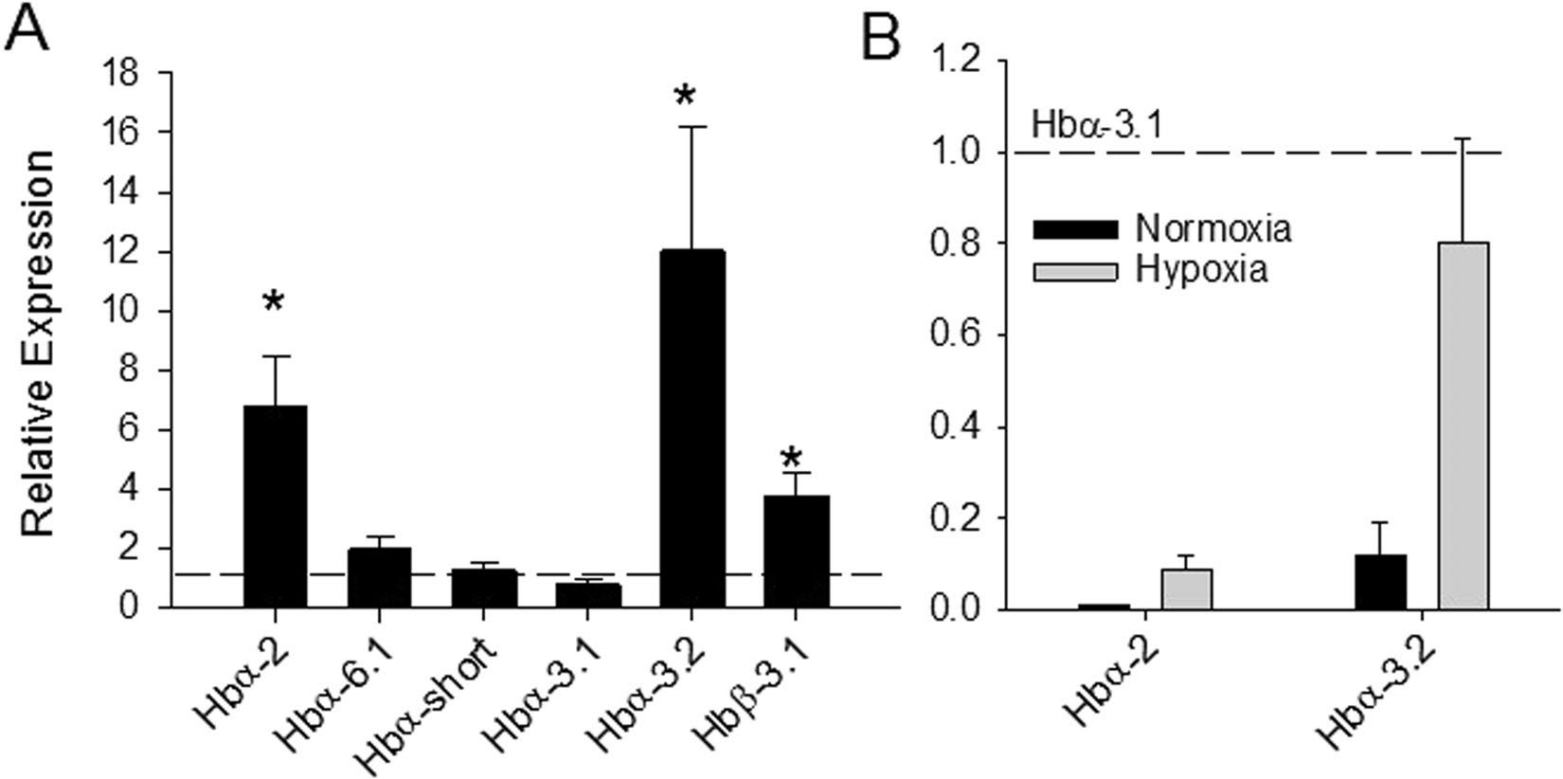
The effect of three-week hypoxia acclimation ($P{{\rm{O}}}_{2}=30 \% \pm 5 \%$) on gene expression of Hbα and Hbβ subunits in the red blood cells of *S*. *ocellatus* as detected by real-time RT-PCR. Panel A demonstrates the effect of hypoxia on relative expression of each individual gene with the dotted line representing the normoxic control value (1). A significant difference between hypoxia and normoxia treatments is denoted by an asterisk (Student’s t-test, P < 0.05). Panel B demonstrates the relative abundance of Hbα-2 and Hbα-3.2 in relation to the predominantly expressed Hbα-3.1 in normoxic and hypoxic acclimations. For all analysis ef1α served as an internal control. All values are mean ± S.E.M; *N* = 7.

Prolonged hypoxia exposure resulted in significant up-regulations of Hb subunits in an isoform specific manner (Fig. 3). Expression levels for Hbα-5, Hbα-6.2 and Hbβ-2 were too low for accurate measurement in both normoxia and hypoxia treatment groups and thus were omitted from further analyses. The relative expression of only three of the twelve gene transcripts increased in response to hypoxia exposure – Hbα-3.2, Hbα-2, and Hbβ-3.1 (Fig. 3A). Importantly, this differential expression response among Hb subunits also resulted in a shift in the predominant Hbα transcripts. Normoxia acclimated fish contained Hbα-3.1 at levels almost 10-times that of the second most abundant isoform, Hbα-3.2 (Fig. 3B). When acclimated to hypoxia, the relative abundance of these two transcripts was not significantly different (one-sample t-test; *P* = 0.43). With respect to the β subunits, Hbβ-3.1 was the most abundant in both normoxia and hypoxia treatments, with expression levels more than two orders of magnitude higher than the other β subunits (data not shown).

### Oxygen transport characteristics

**Figure 4:**
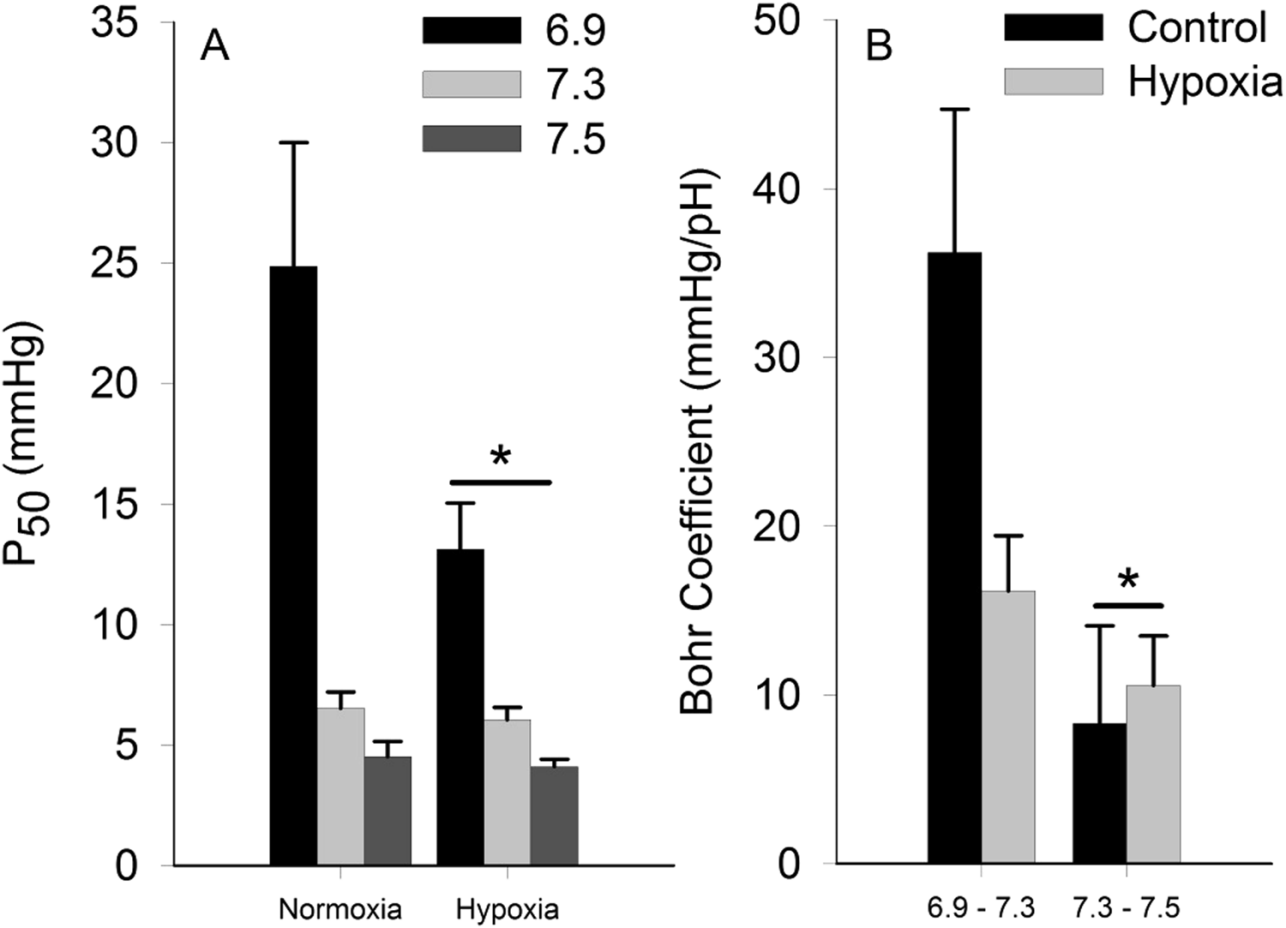
Non-stripped hemolysate P_50_ (**A**) and Bohr coefficients (**B**) for fish acclimated for 21 days to normoxia and hypoxia. A significant difference between treatments is denoted by an asterisk (two-way ANOVA; P < 0.05; *N* = 4–7). Note that a significant interaction between oxygen level and pH was not observed for either endpoint.

**Figure 5:**
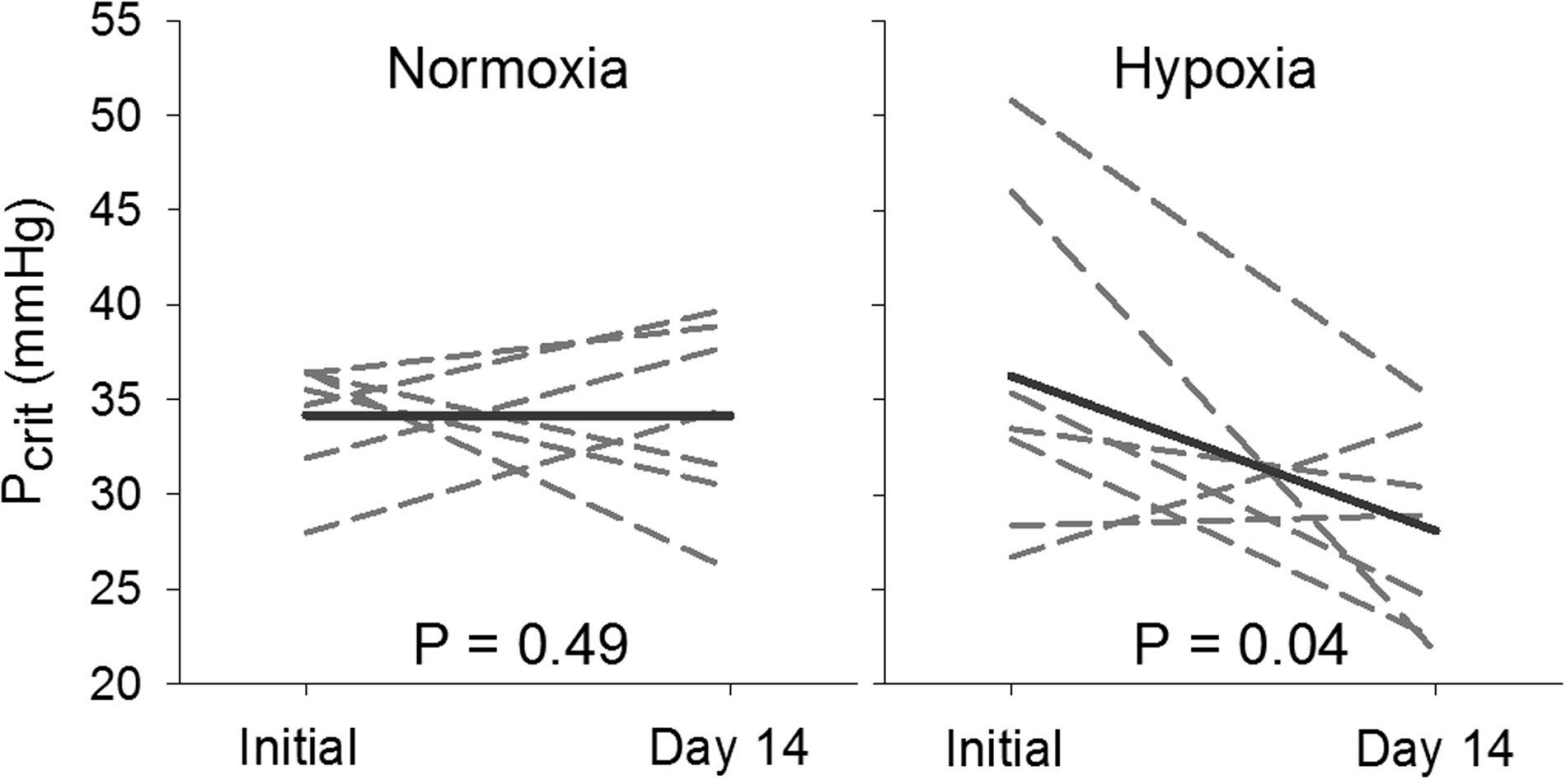
Critical oxygen threshold (P_crit_) of fish before and after a 14 day acclimation to normoxia (**A**) and hypoxia (**B**). Dashed lines represent individual fish and solid lines represent the mean of fish within the respective treatments.

At the red blood cell level, the P_50_ for non-stripped hemolysates was significantly lower (P < 0.05) in hypoxia acclimated fish compared to the normoxia acclimated fish. Note that there was no interaction between pH and hypoxia on P_50_. Bohr coefficients were not different between the two treatments (Fig. 4). At the whole animal level, fish subjected to hypoxia acclimation exhibited a significant 22% decrease (P < 0.05) in P_crit_, from 36.2 ± 3.3 to 28.1 ± 2.1 mmHg. No changes in P_crit_ were observed for fish maintained in normoxia (Fig. 5), and no changes in SMR were observed in either treatment (Table 1).

**Table 1:** Standard metabolic rate (SMR) of fish before and after a 14 day acclimation to normoxia and hypoxia. All data are means ± SEM.

	Standard Metabolic Rate (mgO_2_ kg^−1^ h^−1^)		
	Pre-acclimation	Post-acclimation	P-value
Normoxia	151 ± 8	162 ± 10	0.53
Hypoxia	167 ± 10	166 ± 15	0.97

P-value is determined based on paired t-tests (*N* = 7).

### Morphological characteristics

The total gill surface area standardized for fish mass, as well as the lamellar blood-to-water diffusion distance showed no difference between groups (Table 2). Similarly, hematocrit and relative ventricular mass showed no changes as a consequence of hypoxia acclimation.

**Table 2:** Gill surface area standardized to body mass, diffusion distance, hematocrit, and ventricle weight of *S*. *ocellatus* subjected to 3 weeks of normoxia or hypoxia.

Parameter Measured	Normoxia Group	Hypoxia Group	P-value
Gill Surface Area (mm^2^/g)	$348.9\pm 18.9$	$369.9\pm 24.1$	0.499
Diffusion Distance (μm)	$1.22\pm 0.04$	$1.18\pm 0.03$	0.515
Hematocrit (%)	$37.6\pm 2.4$	$36.6\pm 0.8$	0.706
Ventricle/Body Mass (%)	$7.3\ast {10}^{-4}\pm 3\ast {10}^{-5}$	$7.3\ast {10}^{-4}\pm 3\ast {10}^{-5}$	0.905

No difference was observed. All data are means ± SEM.

## Discussion

The current study sought to explore the ability of red drum – a coastal marine fish native to the Gulf of Mexico – to acclimate to prolonged hypoxia through alterations in blood oxygen transport properties, and the specific role that Hb multiplicity may play in such alterations. The data presented here demonstrate that red drum are able to significantly increase the oxygen binding affinity of Hb in their blood after a three week acclimation period. This is correlated with a change in transcript abundance of specific Hbα subunit isoforms, suggesting a physiological role for Hb multiplicity in this species. These changes in blood oxygen binding affinity are also correlated with a reduction in whole animal critical oxygen threshold. These data strongly suggest that red drum are capable of enhancing their performance in hypoxia through acclimation, which is in part related to phenotypic plasticity of the Hb system.

### Hemoglobin subunit multiplicity

The red drum transcriptome contained 7 different Hbα subunits and 5 Hbβ subunits, and it was confirmed that all of these genes were expressed in sub-adult red drum red blood cells using PCR. While it is difficult to extrapolate transcriptomic information to functional Hb isoforms, owing to the unknown subunit combinations, it is clear that only two subunits (Hbα-3.1 and Hbβ-3.1) account for the majority of observed expression in normoxia. Both of these subunit isoforms have theoretical isoelectric points above 8.0 (data not shown), which indicates that the primary Hb isoforms in red drum are cathodal. However, this should be viewed with caution as these are only theoretical subunit isoelectric points based on amino acid composition.

The currently accepted phylogeny for Hb subunits in fish suggests that the α-subunits are divided among five distinct clades, while there are four β-subunit clades^1, 24, 25^. Our Hbβ phylogeny conforms to this paradigm, but our analysis of the Hbα subunits has both expanded on the phylogeny and provided new insight into the diversity of subunits across different fish lineages. The Hbα phylogeny identified all previously described groups (I-IV and short), but also identified a distinct group (clade V) closely related to clade II. Also of note was the loose collection of sequences with unresolved identities, which included two red drum sequences. We have tentatively named these sequences Hbα-6.1 and -6.2; however, this group may become more resolved as sequence information from additional species becomes available. Several other trends became apparent when the species taxonomy within the respective clades was considered. Only clade III and the “short” clade appear to be widely found among teleosts. Conversely, clade I, II, IV and V are clearly not found across all teleost lineages, with clades I and IV not found in red drum or any other Acanthopterygii. As such, it is important to consider species phylogeny when attempting to understand the functional significance of Hb multiplicity. Interestingly, the most highly expressed Hbα subunits in red drum were both from clade III, which contains isoforms from all tested fish lineages. This may suggest a primary functional role of this particular group of subunits, while other subunits offer specialized or redundant functions.

The Hbα-short isoform was also found to be widespread across fish lineages, but was only expressed at very low levels in sub-adult red drum red blood cells. The conservation of this truncated subunit in all fishes suggests an important physiological role; however, its low expression indicates it isn’t critical for oxygen delivery in developed fish under normal conditions. It is possible that this subunit is important at an earlier life stage; however, currently accepted phylogenetic relationships and the available evidence from rainbow trout suggests that the embryonic subunits are a duplication of Hbα-1^26, 27^.

### Effects of hypoxia acclimation

The P_50_ of red drum non-stripped hemolysates fell within the range typically found in teleosts in both normoxia and hypoxia treatments; however, prolonged hypoxia acclimation was associated with a significantly lower non-stripped hemolysate P_50_ (i.e. increased Hb-O_2_ affinity). The greatest reduction was observed at lower pH; however, the interaction between P_50_ and pH was not statistically significant. The Bohr coefficient for Hb was also not impacted by hypoxia acclimation for either pH range. Interestingly, work on cichlids showed the strongest effects of hypoxia acclimation on P_50_ at pH ≥ 7.6 for both stripped and NTP saturated lysates. While our data were derived from non-stripped lysates, to preserve any hypoxia driven changes in NTP content, the magnitude of observed changes between studies is comparable, with Rutjes *et al*.^5^ reporting hypoxia driven reductions of 1 to 4 mmHg for stripped and saturated lysates. But it is important to remember that P_50_ values from hemolysates are generally lower than intact red cells^28^. This is owed in part to dilution of hemoglobin, NTP and other allosteric modifiers, although the ratio of these parameters remained consistent. Nonetheless, these data give strong evidence that hypoxia acclimation results in an increased blood P_50_ in red drum, which should increase the ability of these animals to extract oxygen from the environment.

A number of factors are known to impact oxygen affinity in fish. These include pH, temperature and Hb concentration, as well as allosteric modifiers such as NTPs^8, 29^. Unfortunately, limitations on sample volume precluded measurement of NTP concentration in our experiment. We instead chose to explore the hypothesis that differential regulation of specific Hb components may coincide with increased oxygen affinity during hypoxia acclimation. It has been previously hypothesized that species with multiple Hbs acting in concert would be better suited to tolerate environmental perturbation^1, 30–32^; however, the physiological support for this is mixed^6, 7, 10, 30^. But previous work has primarily focused on temperature, with relatively less work focusing on prolonged hypoxia acclimation^3, 5, 33, 34^. Here we provide evidence that specific Hb isoforms are selectively up-regulated in response to hypoxia stress, which is also correlated with a decrease in hemolysate P_50_. It is tempting to suggest that the selective up-regulation is promoting an isoHb with higher oxygen affinity, but this cannot be concluded without additional studies that involve purification and protein sequencing (e.g. ref. 35), or *in vitro* translation (e.g. ref. 36). Future work is required to more fully address this question. Nonetheless, our data provide compelling support for the hypothesis that Hb multiplicity provides a physiological benefit to environmental stress, and thus is a phenomenon of evolutionary significance.

An increase in Hb oxygen affinity can play an important role in the ability of fish to maintain oxygen uptake, and aerobic metabolism, as ambient oxygen levels decline (see review ref. 37). In normoxia, the countercurrent arrangement of blood and water flow through the gills will fully saturate blood Hb following gill transit. As ambient oxygen declines, the ability of convective processes to fully saturate blood Hb are constrained, and the degree to which oxygen uptake becomes compromised is largely a product of Hb P_50_. In fact, the minimum partial pressure of oxygen required to maintain standard metabolic rate through aerobic processes (P_crit_) has been linked to whole blood Hb-O_2_ binding affinity^38^. Interestingly, red drum exhibited a significant 22% decrease in P_crit_, from 36.2 ± 3.3 to 28.1 ± 2.1 mmHg, after hypoxia acclimation. Normoxia exposed fish showed no reduction over the same time period. Also note that there was no effect of hypoxia acclimation on SMR, which has also been correlated to P_crit_ in some fish species^39^, although not red drum^40^. Similar findings in response to hypoxia exposure have been shown in goldfish and southern catfish (*Silurus meridionalis*)^41, 42^, with goldfish capable of decreasing P_crit_ by 50% following hypoxia acclimation^41^. It is important to stress that even small reductions in P_crit_ are of substantial ecological importance. When animals are exposed to ambient oxygen levels below P_crit_ they must augment aerobic metabolism with anaerobic metabolism. While animals can vary substantially in their ability to survive on anaerobic metabolism, this strategy is inherently unsustainable over prolonged time scales. By lowering P_crit_, red drum reduce the risk associated with hypoxic environments. Based on our previous work, the 48 mmHg P_O2_ treatment used in this study equates to an approximately 90% reduction in aerobic scope^43^. It would be interesting to assess whether acclimation also increases the available aerobic scope to animals in hypoxia.

Previous studies have shown that a decrease in P_crit_ could be linked to changes in gill morphology^41, 44^, such as an increase in gill surface area or a decrease in lamellar thickness to facilitate oxygen uptake. In red drum, gill surface area and lamellar thickness were not different between treatments. Thus gill remodeling does not account for the altered P_crit_ in red drum. Similarly, hematocrit was not different between normoxia and hypoxia acclimated individuals, which is a factor that has also been shown to maximize blood oxygen-carrying capacity in response to hypoxia^44, 45^. Finally, increased heart mass as a consequence of hypoxia exposure can also impact oxygen transport properties^46^, but again no differences in relative ventricular mass were observed between treatments. Thus it is likely that the observed changes in P_crit_ are related specifically to the changes in Hb oxygen binding affinity. Our data are also consistent with the hypothesis that the observed change in Hb oxygen binding affinity is related, at least in part, to differential regulation of Hb subunit isoforms. Unfortunately, the inability to assess changes in red blood cell NTP levels make this conclusion somewhat ambiguous.

### Conclusion

The data presented here provide support for the hypothesis that marine fish endemic to hypoxia prone environments are capable of enhancing their performance through acclimation. In the case of red drum, the available evidence suggests the acclimation response was limited to the Hb system, with no changes in branchial characteristics, hematocrit or relative ventricular mass. Differential regulation of specific Hb subunit isoforms coincident with the reduction of hemolysate P_50_ is consistent with the theory that Hb multiplicity in fish provides an adaptive advantage when confronted with environmental challenges, either through the expression of functionally distinct isoHbs or the presence of distinct environmentally triggered transcriptional response elements that allow a greater level of regulation. Importantly, these gene and organ level responses also manifested at the whole animal level through a significant reduction in P_crit_. Overall, the results of this study demonstrate that the red drum is capable of enhancing aerobic performance under hypoxia through acclimation.

## Methods

### Phylogenetic analysis

All analyses were performed using the freeware MEGA version 6.06 software package. Alignments were performed on putative amino acid sequences using the ClustalW function and the Gonnet protein weight matrix. Sequences were first identified using a combination of a Blast search of the NCBI database as well as a targeted selection based on literature. Preliminary analyses of all identified sequences were performed using the neighbor joining method, after which the sequences used for analysis were pruned to limit species specific duplication events (excluding red drum). This was performed to simplify analysis and graphical display to emphasize deeper phylogenetic trends. Similarly, targeted Blast search efforts were made to ensure proper coverage of the Acanthopterygii, Protacanthopterygii, and Otocephala superorders across the various Hb subunit isoforms. Final analyses were performed using maximum likelihood methods on protein sequences with the following options: JTT model, NNI ML heuristic method and complete deletion of gaps/missing data. Phylogenies were tested using bootstrap methods with 1000 iterations. All sequence identifiers within the final analysis are included in Fig. 1.

### Experimental fish

All experimental procedures were approved by the University of Texas at Austin Institutional Animal Care and Use Committee (IACUC), and all experiments were performed in accordance with the approved IACUC protocols and methodologies. Unless specified all chemicals were obtained from Fisher Scientific. Red drum were obtained from Copper Shoals Red Drum aquaculture facility (El Campo, Texas), and held on-site at the University of Texas Fisheries and Mariculture Laboratory. Sub-adult red drum (91 ± 5 g; mean ± SEM) were held in recirculating in-door 150 L tanks supplied with filtered natural seawater maintained at 22 °C. The recirculation system was equipped with a common biological filter tank to control ammonia levels. Fish were fed daily with commercial fish pellets (Aquafeed, Cargill, USA) and tanks were siphoned daily to remove debris. All fish were acclimated to the laboratory setting for at least two weeks prior to experimentation.

### P_crit_ measurement

Food was withheld for at least 48 h prior to measurements. Standard metabolic rate (SMR) of fish was measured in advance of P_crit_ measurement. Oxygen consumption rate ($\dot{{\rm{M}}}$O_2_) of the fish was measured using computerized intermittent-flow respirometry (Loligo Systems, Denmark) for SMR calculations^47^ at 24 °C for at least 20 h. Each $\dot{{\rm{M}}}$O_2_ measurement cycle lasted 6 min, consisting of a 3 min flushing period followed by a 3 min closed period. $\dot{{\rm{M}}}$O_2_ was calculated from the decline in PO_2_ in the last 2 min of the closed period according to Eqn 1:

1 $${\dot{{\rm{M}}}O}_{2}=\frac{-{{\rm{\delta }}\mathrm{PO}}_{2}\times {\rm{\alpha }}{O}_{2}{{\rm{H}}}_{2}{\rm{O}}\times ({{\rm{V}}}_{chamber}-{{\rm{M}}}_{f})}{{{\rm{M}}}_{f}}$$

where $\dot{{\rm{M}}}$O_2_ is oxygen uptake (μmol kg^−1^ min^−1^), δPO_2_ is the slope of the decline in water oxygen tension (mmHg min^−1^) during a closed respirometer cycle, αO_2_H_2_O is the solubility of oxygen in the water at the relevant temperature (μmol L^−1^ mmHg^−1^), V_chamber_ is the volume of the respirometer (L), and M_f_ is the mass of the fish (kg). Values for $\dot{{\rm{M}}}$O_2_ were omitted when the R^2^ of the linear regression for the decline in PO_2_ was less than 0.95. Background tests were conducted for 0.5 h-2 h both before the fish was placed into the respirometer chamber and after the fish was removed from the respirometer chamber. Background $\dot{{\rm{M}}}$O_2_ was subtracted from the calculated $\dot{{\rm{M}}}$O_2_, by assuming a linear increase in O_2_ consumption rate over the measurement interval. The lowest 10% $\dot{{\rm{M}}}$O_2_ values obtained from the measurement period was averaged to determine SMR for comparison with previous work conducted in our lab on red drum^40^. Directly following SMR measurement, the fish was subjected to a P_crit_ trial whereby the respirometer was placed on a closed circuit until P_O2_ within the chamber dropped below 8% of air saturation (12.7 mmHg). $\dot{{\rm{M}}}$O_2_ was calculated from the decline in P_O2_ in 2 min intervals during the closed period. P_crit_ was then determined as the P_O2_ where $\dot{{\rm{M}}}$O_2_ was reduced below the SMR^40, 43^.

### Hypoxia acclimation experiment

Exposures were performed in two 150 L tanks, with one maintained at hypoxic condition (P_O2_ = 48 ± 8 mmHg) and the other at normoxic condition (P_O2_ ≥ 151 mmHg). Hypoxia was maintained using an automated nitrogen bubbling system, which consisted of a fiber optic oxygen electrode and associated meter (PreSens) interfaced to a gas flow solenoid, which was controlled using AutoResp software (Loligo Systems). Tanks were monitored daily for salinity and pH, and temperature in both tanks was maintained at 22 °C. Fish were sub-dermally tagged with VI alpha tags (Northwest Marine Technology, USA), according to the manufacturer guidelines, 1 week prior to experimentation. Individuals were then randomly assigned to either hypoxia or normoxia treatments, with 7 fish per treatment. P_crit_ of all experimental fish was measured before subjecting them to their respective treatments and at the end of the 2-week acclimation period. Fish were fed *ad libitum*. After the final P_crit_ measurement, fish were placed back into their respective treatments for 1 week to recover from the P_crit_ test, after which individuals were euthanized and sampled as described below.

### Sampling and analysis techniques

Fish were euthanized by full immersion in a MS-222 bath (250 mg/L; 500 mg/L NaHCO_3_) followed by spinal transection. Body mass was recorded, and blood was taken by caudal puncture using a 23-gauge needle pre-rinsed in heparinized saline (50 units/mL). Hematocrit was measured and blood samples were centrifuged at 13,000 rcf for 1 min to separate the plasma. Packed red blood cells were preserved at −80 °C for further analysis. The heart was excised and the ventricle was separated and weighed. The first gill arch on the left side of the fish was sampled for gill surface area and gas diffusion distance analysis. Gill samples were placed immediately in zinc formalin fixative (Z-Fix; Anatech Ltd, USA), stored overnight, and subsequently transferred to 70% ethanol for long-term storage.

### RNA analysis

Total RNA was extracted using TRI Reagent (Molecular Research Center Inc., USA) according to manufacturer guidelines, with homogenization performed using a 18 gauge and then a 23 gauge needle. Total RNA was quantified using an ND-1000 (Thermo Scientific, USA) spectrophotometer at a wavelength of 260 nm and sample purity was assessed using 260:280 ratios. cDNA synthesis was performed using RevertAid reverse transcriptase (Thermo Scientific) according to the manufacturer specification, using 1 μg of DNase I (Thermo Scientific) treated total RNA as template. Relevant Hbα and Hbβ gene sequences were identified from a commercially developed (LC Sciences, USA) RNA-Seq transcriptome library for red drum. Primer sets were designed using Primer3^48, 49^ and specificity of each set was verified using standard PCR and gel electrophoresis procedures. All primers are listed in Table 3. Real-time PCR was performed on an Mx3000 P real-time PCR system (Stratagene, USA) using the Maxima SYBR green master mix kit (Thermo Scientific; 12.5 μL reactions). Thermocycler program and reaction composition were optimized for the highest PCR efficiency calculated using a cDNA standard curve (94 °C 15 s, 60 °C 30 s, 72 °C 30 s, 40 cycles; 0.3 μM primer pair), and primer specificity was accessed using the disassociation curve of each reaction. PCR efficiencies ranged from 77 to 96% with an R^2^ ≥ 0.99. Relative mRNA expression was calculated using the delta-delta ct method using elongation factor 1α (ef1α) as an internal control^50^. The relative abundance of Hbα transcripts within each sample was also assessed using a rearrangement of the numerator from the delta-delta ct formula, which allowed proper incorporation of the respective PCR efficiencies (*E*_Hbα-3.1_^ct^/*E*_n_^ct^). In this case, relative abundance was calculated relative to the dominant transcript in normoxia, Hbα-3.1. Successful DNase treatment was verified using a no reverse transcriptase control for each treatment.

**Table 3:** List of real-time PCR primers used for Hb subunit gene expression analysis.

Gene	Orientation	Sequence
EF1α	F	GTT GCT GGA TGT CCT GCA CG
	R	GTC CGT GAC ATG AGG CAG ACT G
Hbα-2	F	TTT CAG GTG CTG TGA GAG AGA G
	R	GCC AGA GTT TTG ACT CAG GTC T
Hbα-6.1	F	AAA TAC CGA TAA ACT GCA AAC AGG
	R	AGA GTA TCC GAG CTT TTG GTA TTG
Hbα-Short	F	ATG CTC TCA AAG AAG GAG AAA GAG
	R	GAT GGG AAA AGT ATG TTT TTG TGC
Hbα-3.1	F	GTA GGT GCT TCT TCC CCA CA
	R	CTT AAG CCA CCG ACA AGG TC
Hbα-3.2	F	TAA TCT TGT CGG TGC TAT GAA GG
	R	CCA GGG AAG TAC ATG CTG ATT AC
Hbα-5	F	TAA ACA GCA GGA GAA GAT GAT GG
	R	CGT TTT TGC ATT CAT GTG TTT AT
Hbβ-1	F	AAA GTT GGG TAA AGC CTT CAC TG
	R	GTC TTC TGT TGC AGC TTT CTA GTG
Hbβ-4	F	GCT GTT TGG GAA AAG GTT GTA A
	R	TAT CCC CAA AAC TTC CGA AAT A
Hbβ-2	F	AAC TCT TCA TCT CCA GCC TAT CAC
	R	TGC CAA AGA TCT TGG TGA TGA T
Hbβ-3.2	F	GCT TGC TAT CAG AGA ACT CGT TTG
	R	TGT TGA TGG TGT GAA AGT CTT CTT
Hbβ-3.1	F	TTA ATA AAA GCC TCC AAA GGA CTG
	R	CAT GTT GAC GAG GTT TAG GTT TAA G

All sequences listed 5′ to 3′ with the reverse primer sequences listed as the reverse compliment of the gene sequence.

### Hemoglobin Oxygen Affinity

All Hb-O_2_ equilibria experiments were performed at the University of British Columbia (UBC), Vancouver, Canada. Isolated red blood cells were delivered to UBC on dry ice and subsequently stored at −80 °C until use. For analysis, red blood cells were thawed on ice and the Hb concentration was quantified using the Drabkin’s method^51^. Note that owing to the small sample volume (50–80 µl) the sample was mixed with 40 µl of deionized water prior to quantification, which both maximized sample utility and pipetting accuracy during quantification. Methemoglobin concentration was assessed spectrophotometrically and used to correct total Hb concentration to ensure a functional Hb_4_ concentration of 0.6 mM. There was no significant difference in methemoglobin levels between control and hypoxia acclimated fish. Hemolysates were prepared in HEPES buffer (100 mM) of nominal pH 7, 7.4 and 7.8 supplemented with 40 mM KCl and 4 mM ATP. Hb-O_2_ equilibria were constructed using a previously described custom built gas equilibration chamber^52^ placed within a plate spectrophotometer (Molecular Devices). Gas flow was controlled by a Wösthoff DIGAMIX® gas mixing pump (©H. Wösthoff Messtechnik, Bochum, Germany). Assays consisted of equilibration at 0, 0.05, 0.1, 0.15, 0.25, 0.5, 0.75, 1, 1.5, 2, 5, 8, 12, 21 and 100% O_2_ balanced with N_2_, and the absorbance plateaus at each O_2_ tension were assessed manually by the operator. All assays were performed at 22 °C. The absorbance at each O_2_ tension was used to generate Hill plots, log([oxyHb]/[deoxyHb]) vs logPO_2_, and thereby estimate P_50_ – the partial pressure that results in 50% Hb-O_2_ saturation. Each plot was assessed manually to verify proper dose response relationships with respect to absorbance and O_2_ level. P_50_ was estimated from Hill plots using the following formula: ${\rm{P}}50={10}^{(-\frac{intercept}{slope})}$. The pH of each diluted hemolysate was assessed using a microelectrode system, which was used for calculation of Bohr coefficients.

### Gill surface area and gas diffusion distance

Measurements of gill surface area were carried out according to methods outlined by Hughes^53^. Briefly, the total number of filaments on the first gill arch was counted and the length of every tenth filament was measured under a dissecting microscope. The linear spacing between lamellae along the filament was measured along at least 8 lamellae at 100X magnification under a light microscope (Nikon Eclipse TE2000-U) at the base, midsection and tip of 5 filaments evenly distributed along the gill arch using digital image capture software (QED Capture). An image of a stage micrometer was also digitally captured to calibrate the measurements.

Approximately 10 filaments from the midsection of the gill arch were dehydrated first in an ethanol series (95%, 3*60 min; 100%, 3*45 min), then in butanol for 1 h, and finally left in butanol overnight (12–16 h). Filaments were then treated twice with Histochoice clearing reagent (Amresco, USA) for 90 min, followed by two 60 min washes in Paraplast Plus embedding media (Leica Biosystems Richmond Inc., USA) at 58 °C. The samples were embedded in casting cubes at room temperature and stored at 4 °C until sectioned. Samples were sectioned at 6 μm using a microtome and mounted onto Superfrost Plus slides (VWR, USA). At least 5 sections were mounted onto each slide. Slides were then deparaffinized with two 5 min washes in Histochoice clearing agent and rehydrated in an ethanol series (100%, 2*5 min; 95%, 1*5 min; 70%, 1*5 min). Slides were stained with hematoxylin solution, gill no. 2 (Sigma-Aldrich, USA) for 120 s, soaked in 0.3% HCl-EtOH solution for 60 s, blued in Scott’s tap water substitute (RICCA Chemical, USA) for 45 s and counterstained with eosin y solution, alcoholic (Sigma-Aldrich) for 60 s. Slides were rinsed with tap water in between the different solutions and soaked additionally for 30 s in 95% ethanol before counterstaining. Coverslips were then applied with Permount^®^ as a mounting medium after the slides were dehydrated.

Stained slides were viewed under a light microscope (Nikon Eclipse TE2000-U) at 400X magnification. Digital images of 20 lamellae were captured for each fish to quantify lamellar area. All lamellae were selected from the midsection of the filament and no more than 4 lamellae were chosen from a single filament. Images were assigned random numbers before surface areas were scored to minimize bias. Total surface area was calculated as $A=LfB$ where *L* is the total filament length (mm), *f* is the number of lamellae per millimeter on both sides of the filament, and *B* is the average bilateral surface area of a single lamella (mm^2^). Surface area was standardized to the mass of the fish for comparisons between different fish.

Fifteen digital images of lamellae were then taken for each fish under 1000X magnification and 10 measurements of lamellar blood-to-water diffusion distance were taken to estimate the lamellar blood-to-water diffusion distance of each fish. Similar to surface area measurements, images were also assigned random numbers before diffusion distances were scored.

### Statistical Analyses

Data are presented as means ± SE. Gene expression levels and morphometric data collected after the acclimation experiment were analyzed using unpaired t-test to identify differences between the normoxia and hypoxia group. Statistical significance was assumed at P < 0.05. Blood P_50_ data was analyzed using a two-way ANOVA with pH and acclimation condition as the two independent factors. Note that a significant interaction between factors was not observed, thus treatment effects at a specific pH could not be assessed. P_crit_ from hypoxia acclimation experiment was analyzed using one-tailed paired t-test, under the *a priori* assumption that dynamic Hb expression and reduced hemolysate P_50_ should reduce P_crit_.
